# The effects of fresh embryo transfers and elective frozen/thawed embryo transfers on pregancy outcomes in poor ovarian responders as defined by the Bologna criteria

**DOI:** 10.4274/tjod.76402

**Published:** 2015-09-15

**Authors:** Serdar Çelik, Niyazi Emre Turgut, Erbil Yağmur, Kübra Boynukalın, Dilek Cengiz Çelik, Necati Fındıklı, Sevim Purisa, Mustafa Bahçeci

**Affiliations:** 1 Bahçeci Fulya in Vitro Fertilization Center, İstanbul, Turkey; 2 İstanbul University Faculty of Medicine, Department of Biostatistics and Medical Informatics, İstanbul, Turkey

**Keywords:** Poor responder, Frozen embryo transfer, endometrial receptivity

## Abstract

**Objective:**

To compare the effects of fresh embryo transfers (ET) and elective frozen/thawed embryo transfers (eFET) on implantation, clinical pregnancy, and live birth rates in poor ovarian responders, as defined by the Bologna criteria.

**Materials and Methods:**

All electronic databases of embryo transfers between January 2011 and January 2014 were retrospectively reviewed. Two hundred fifty-nine of all the fresh ET and 96 of all eFET were included into the study. An antagonist protocol with letrozole was used for the controlled ovarian hyperstimulation (COH) in all participants.

**Results:**

The mean age was 36.9 years (range, 21-43 years) in the fresh ET arm and 37.2 years (range, 21-43 years) in the eFET arm (p=0.45). The clinical pregnancy rate was 35% (90/259) versus 29% (28/96); the abortion rate was 27% (20/75) versus 36% (9/25); and the live birth rate was 21% (55/259) versus 17% (16/99). There were no significant differences between groups and p values were 0.32, 0.52, and 0.42, respectively. The mean E2 level was 389 (range, 50-2055 pg/mL) in the fresh ET group (on hCG day) and 418 pg/mL (range, 121-3073 pg/mL) in the eFET group (on day 14 of cycle) (p=0.122). No differences were found between the two groups with respect to the total number of retrieved oocytes (p=0.55) and number of metaphase II (MII) oocytes (p=0.81). The number of embryo transfers was statistically different (p=0.005). The effects of age, total number of retrieved oocytes, number of MII oocytes, type of treatment, number of ET, and the day of ET and E2 level to live birth outcomes were investigated using binary logistic regresion analyses, and no stastical effect was determined by any of the parameters. P values were p=0.50, 0.66, 0.45, 0.30, 0.30, 0.08, and 0.90, respectively.

**Conclusion:**

E2 levels tend to be lower in poor responders, thus the receptivity of the endometrium may be damaged less than normal, which may explain why pregnancy results are the same between eFET and ET groups.

## INTRODUCTION

The success rates among infertility treatments are steadily increasing with the new technological developments in the multiple oocyte collecting process through controlled ovarian hyperstimulation (COH) and cryopreservation methods. However, pregnancy rates in patients who are so-called poor responders are still low^(1,3)^.

For a successful implantation, there has to be a good quality embryo as well as a normal receptive endometrium. In natural cycles, endometrial receptivity arises 6 to 10 days after ovulation and this period is called the “implantation window”^(4)^. The quality of receptivity in this time frame plays an important role in in vitro fertilisation/ intracytoplasmic sperm injection (IVF/ICSI) failures^(5)^. In COH cycles, endometrial receptivity is negatively effected because of the supraphysiologic hormonal environment^(6,7,8,9)^.

The definition of poor ovarian response varies in the literature. Some of the criteria used to define poor resonders are serum E2 levels less than 500 pg/mL;^(10)^ antral follicle count (AFC) less than 3;^(11)^ retrieval of less than 3 oocytes in a previous situmulation protocol;^(12)^ and the level of day 3 follicle stimulating hormone (FSH) >12 mIU/mL^(13)^. Recently, Ferraretti et al.^(14)^ published their detailed poor-responder definition.

The aim of this study was to compare the effects of elective frozen/thawed (eFET) and fresh embryo transfers (ET) on implantation rates, clinical pregnancy, and live birth outcomes in poor ovarian responders, as defined by the Bologna criteria.

## MATERIALS AND METHODS

This study was carried out in Bahçeci Fulya IVF Center, İstanbul. The records were retrospectively reviewed and as such there was no need for ethical committee approval.

All electronic records of embryo transfers between January 2011 and January 2014 were analyzed. Overall, 14 601 patients were reviewed. Two hundred fifty-nine patients comprised the fresh ET arm and 96 were included the eFET arm of the study.

To ensure homogeneity between groups and show the effectiveness of embryo transfers, exclusion criteria were carefully generated as follows;

i) AFC ≥6-8

ii) 4≥oocytes retrieved in previous COH

iii) Aged over 43 years on the day of embryo transfer

iv) Dehydroepiandrosterone (DHEA) or growth hormone (GH) therapy before COH

v) The COH protocols of those who did not use the letrozole/antagonist regime

vi) History of difficult embryo transfer and use of a hard catheter (Wallace Sure-Pro, Two-Stage set, UK)

vii) Progesteron level higher than 1.5 ng/mL on the human chorionic gonadotropin (hCG) day for fresh EET cycles, and on day 14 of treatment for the eFET cycles.

All patients who met the definition of Ferraretti et al.^(14)^ as poor responders were included in the study.

Second embryo transfers with remaining frozen/thawed embryos in the eFET arm were excluded from present study.

Frozen/thawed blastocysts were transfered without undergoing preimplantation genetic tests for aneuploidy screening or other anomalies. All patients used their own oocytes because donor oocytes are strictly forbidden in Turkey.

To evaluate the endometrial receptivity, E2 levels were measured on day 14 of the cycle for the eFET arm and on hCG day for the fresh ET arm.

### Stimulation protocol

Letrozole (Femara; Novartis, İstanbul, Turkey) 2.5 mg was started twice daily on day 2 of the current cycle and used for 5 days. Human menopausal gonadotropin (hMG) 300-450 IU (Merional; IBSA Institut Biochimique SA Lamone, Switzerland) was initiated on day 7 of the cycle and ovarian response was evaluated using transvaginal sonography and serum E2 levels. The gonadotropin-releasing hormone antagonist (Cetrotide; Serono, Turkey) was administered when the leading follicle reached 11-13 mm and used until to final triggering. Two ampoules of recombinant hCG (Ovitrelle; Serono, Turkey) was administered as soon as the leading follicle reached a mean diameter of 18 mm. We believe we can pick up more mature oocytes using 500 µg recombinant hCG in patients who are poor responders^(15)^. The oocyte pick-up procedure was performed 34-36 hours after recombinant hCG injection using transvaginal ultrasound-guided needle aspiration under general anesthesia.

### Luteal support

After the oocyte retrieval, vaginal progesteron gel (Crinone 8%, MerckSerono, Bedfordshire, UK) 90 mg was used twice a day^(16)^ until the pregnancy test. For positive test results, luteal phase support was maintained up to the 10^th^ week of pregnancy.

### Endometrial preparation

Transdermal estradiol hemihydrate patches (Climara Forte, Bayer, İstanbul, Turkey) were started on either of day 3 of the following cycle or as 100 mcg/day for the first 4 days of the next cycle, 200 mcg/day for the following 4 days, and 300 mcg/day for the last 4 days. Serum progesterone level and endometrial thickness were then measured. Treatment was continued if progesterone level <1.5 ng/mL, endometrial thickness >8 mm and had a triple-line appearance. Vaginal progesteron gel (Crinone 8%, MerckSerono, Bedfordshire, UK) 90 mg was prescribed twice a day. A transdermal estradiol hemihydrate patch 100 mcg/day and vaginal progesterone gel at the same dosage were maintained until the pregnancy test. For positive test results, patients used vaginal progesterone up to the 10^th^ week of pregnancy.

### Protocol for vitrification and embryo-thawing

We prepared our own solutions for embryo vitrification and thawing procedures. Day 2-3 embryos and day 5-6 embryos were put in an equilibration solution at room temperature for 6-8 minutes and 10-12 minutes, respectively. They were then kept in the vitrification solution for 40 seconds and directly submerged into liquid nitrogen after loading into the cryovial.

For thawing, the cryovial was taken out of the liquid nitrogen, kept in the first thawing solution at 37 °C for 1 minute, in the second thawing solution at room temperature for 3 minutes, and then transferred into the culture solution to be put into the incubator afterwards.

### Pregnancy definitions

Serum hCG levels were evaluated 12 days after embryo transfer. Values >5 mIU/mL were accepted as a positive result.

Clinical pregnancy was defined as an intrauterine sac envisioned using transvaginal sonography at 7 weeks of gestation.

Live birth was defined as birth of one or more infants including a gestational age of ≥20 weeks^(17)^.

Abortion was defined as an unintentional expulsion of an embryo or fetus before the 20^th^ week of gestation.

### Statistical analysis

Pregnancy rates per patient and per ET were calculated by dividing intrauterine gestational sac number by all cohorts and the number of transferred embryos.

Clinical pregnancy rates per patient and per ET were calculated by dividing the number of all women with intrauterine sacs by all cohort and the number of transferred embryos.

Live birth rates per patient and per transferred embryo were calculated by dividing the total number of births at a gestational age of ≥20 weeks by all cohort and transferred embryos.

Abortion rates were obtained by dividing the number of pregnancy losses by the number of clinical pregnancies.

Dichorionic pregnancies were accepted as two gestational sacs, but monochorionic pregnancies as one.

Distribution characteristics of variables were assessed with histograms, Kolmogorov-Smirnov and One-Sample test. Data is presented as median, minimum, maximum, frequency and percentage. Quantitative variables that seemed to affect live birth outcomes were analyzed using the Mann-Whitney U test. Categorical variables were compared using the Chi-Square test. P<.05 was considered to be statistically significant. Logistic regresion analysis was used to investigate factors that affected live birth outcomes. Statistical calculations were performed using the Statistical Package for the Social Sciences (SPSS), version 21.0.

## RESULTS

Three hundred fifty-five women were enrolled into the study. Groups were generated as the fresh ET arm, which included 259 patients, and the eFET transfer arm, comprised by 96 patients.

Age, metaphase II oocyte (MII) distribution, total number of retrieved oocytes, and pregnancy outcomes were similar in both treatment arms (p=0.45, 0.51, 0.55, 0.66, respectively). The mean serum E2 level measured on hCG trigger day in the fresh ET arm was 389 pg/mL (range, 50-2055 pg/mL) and on day 14 of the cycle in the eFET arm it was 418 pg/mL (range, 121-3073 pg/mL). There was no significant difference between groups regarding serum E2 levels (p=0.12). In addition, the mean number of transferred embryos was 1 (range, 1-2) in the fresh ET arm and 2 (range, 1-2) in the eFET arm; this difference was statistically different between the groups (p=0.005) (Table 1).

In the fresh ET arm, 164 (63%) out of 259 transfers were single embryo transfers (SET) and in 51 (53%) out of 96 transfers in the eFET arm were double embryo transfers (DET). There was no significant difference with respect to implantation rates between the groups (p=0.66).

Positive pregnancy test results were obtained in 90/259 (35%) in the fresh ET arm, and in 31/96 (32.3%) in the eFET arm (p=0.66). The number of single gestational sacs was 70/259 (27%) and 22/96 (22%), and double sacs was 10/259 (3.9%) and 3/96 (3.1%), respectively.The clinical pregnancy rates in both groups were 35% (90/259) versus 29% (28/96) and no stastical difference was determined (p=0.32). The number of missed abortions was 20/75 (27%) with fresh ET and 9/25 (36%) with eFET (p=0.52), and the number of biochemical pregnancies was 9/259 (3.5%) and 5/96 (9.4%), respectively. Two ectopic pregnancies were diagnosed in the fresh ET group. The number of live births was 55/259 (21%) in the fresh ET group and 16/96 (17%) in the eFET group; no stastical difference was noted (p=0.34).

There was no significant difference related to pregnancy, clinical pregnancy, and live birth outcomes per number of embryo transfers (p=0.30, 0.13, and 0.17, respectively) (Table 2).

The distrubition of the day of embryo transfers between groups were different. The number of clevage stage embryo transfers in the fresh ET arm was higher than in the eFET arm (250/259 (96.5%); 85/96 (85.4%)), and the number of blastocyst transfers was higher in the eFET arm (14/96 (15%); 9/259 (4%)) (p=0<001).

The effects of total number of retrieved oocytes, number of MII oocytes, number of transferred embryo, day of ET, type of treatment, and age to live birth outcomes were evaluated seperately. Only age was found as an affecting variable. P values were 0.65, 0.30, 0.62, 0.16, 0.42, 0.001, respectively (Table 3).

In addition, we conducted subgroup analyses and found a positive effect of blastocyst transfer on live birth rates. The live birth rate was 32% (13/41) after blastocyst transfer and 19% (58/314) after cleavage stage embryo transfer (p=0.036).

The effects of age, total number of retrieved oocytes, number of MII oocytes, type of treatment, number of ET, the day of ET, and serum E2 to live birth outcomes were investigated using binary logistic regresion analyses. The Nagelkerke R2 value was 0.75, Hosmer-Lemeshow goodness of fit test was p=0.99, and the estimation percentage of model was 89.5%. None of the variables were found as a risk factor in live birth outcomes (p=0.50, 0.66, 0.45, 0.30, 0.30, 0.08, 0.90, respectively). Although age was different in univariate analyses, no difference was found in multivariate analyses.

## DISCUSSION

In this study we found no significant differences regarding clinical pregnancy, abortion, and live birth rates between the eFET and fresh ET groups.

Prostaglandin synthesis is diminished in the early pregnancy period. It has been reported that increased levels of prostaglandins may be a cause of early pregnancy loss^(18,19)^. Prostaglandin levels tend to be higher after oocyte retrieval and this may be the reason for increased numbers of early pregnancy losses in fresh embryo transfer cycles, and also for early ovarian hyperstimulation syndrome (OHSS)^(20,21)^. For this reason, it has been claimed that eFET cycles are similar to natural cycles. However, younger age groups and patients who were normoresponders comprised the cohorts in the aforementioned studies. In the present study, the mean age of the eFET arm was 37.2 years (range, 21-43 years) and 36.9 years (range, 21-43 years) in the fresh ET arm, hence higher abortion rates were observed at 27% and 36% respectively, which were consistent with the literature^(22)^. Oocyte-induced factors have been suggested for these higher and similar abortion rates. Beyond this factor, uterine microenvironments may resemble each other owing to similar levels of estrogen.

It has been argued that the supraphysiologic hormonal microenvironment in fresh ETcycles may negatively affect the implantation process^(20,23)^. COH procedures may change the gene expression profile of endometrium. There may be alterations in E2 and progesteron receptors in stimulated cycles; therefore, endometrium maturation may be negatively affected with respect to normal endometrium^(24)^.

Supraphysiologic serum levels of E2 (>2500 pg/mL) may hamper endometrial maturation and implantation, hence pregnancy rates in these cycles are lower. Reducing serum levels of E2 in the preimplantation period may improve pregnancy outcomes^(25,26,27)^. However, a systematic review and meta-analyses comparing fresh ET and eFET reported higher clinical and pregnancy rates in the eFET group among patients who were normoresponders;^(28)^ no consensus exists for patients who are poor responders.

In the present study, we noted similar serum E2 levels in both arms. During endometrial preparation, the mean serum estradiol level on day 14 was 418 pg/mL (range, 121-3073 pg/mL) in the eFET arm, and 389 pg/mL (range, 50-2055 pg/mL) in the fresh ET arm on the day of hCG trigger (p=0.12). These findings displays a similarity between groups with respect to endometrial receptivity and pregnancy outcomes.

Letrozole, a nonsteroidal reversible aromatase inhibitor, is used in in vitro fertilization cycles among patients who are poor responders. It is thought to inhibit peripheral conversion of androgens to estrogen by blocking aromatization and decreases negative estrogen feed back to the pitutary gland^(29)^. Sequential use of letrozole and gonadotropins has been reported as a better method for follicular growth in poor responders^(30)^. Furthermore, decreased estradiol levels have been documented in letrozole cycles compared with long protocols^(31)^. Several later studies supported these findings^(29,32,33,34)^. Letrozole use in the course of the hyperstimulation period may cause more physiologic endometrial receptivity. In the present study, only patients who used used letrozol/antagonist treatment were included in the fresh ET arm. In this way, we aimed to prevent estradiol increments and provide more physiologic endometrial receptivity as a consequnece. In addition, the diminished antral follicle count may be a reason for decreased estradiol levels in COH procedures among patients who are poor responders^(10)^.

Progesteron is one of the other hormones that disrupts endometrial receptivity^(28)^. Levels higher than 1.5 ng/mL on the day of the hCG trigger negatively affects pregnancy outcomes^(35)^. In our study, progesterone levels above 1.5 ng/mL on the day of hCG trigger were exclusion criteria for the fresh ET arm and on day 14 for the eFET arm. Thus, the potential negative impacts of progesterone on endometrial receptivity was prevented.

Endometrial receptivity may be hampered much less in poor responders because of lower serum E2 levels in COH cycles. In this manner, pregnancy outcomes between groups would be similar. We believe that the decreased pregnancy rates among poor responders are due to oocyte factors.

There is no consensus regarding the ET day in poor responders^(36,37,38,39)^. The disparities in embryo transfer days may not be controlled between the eFET and fresh ET groups due to the retrospective design of our study. Among all patients, 23 (7%) out of 355 were blastocyst transfers and the remainder (332/355; 93%) consisted of cleavage stage embryo transfers. The blastocyst transfer rate was higher in the eFET arm, and the cleavage stage embryo transfer rate was higher in the fresh ET arm (p<0.001). There was no detrimental effect observed regarding the day of embryo transfer on live birth rates, even though the distrubition of transfer days were different in both arms (p=0.08).

Our study was of a retrospective design and did not eliminate confounding factors such as smoking, Body mass index (BMI), duration of infertility, and underlying sistemic diseases. Further large randomized controlled studies are needed because of these limitations.

In conclusion, we found similar pregnancy results among patients who were poor responders in both treatment arms. We believe that similar serum E2 levels and as a result, similar endometrial receptivity patterns might be the main reason for this finding.

## Figures and Tables

**Table 1:**
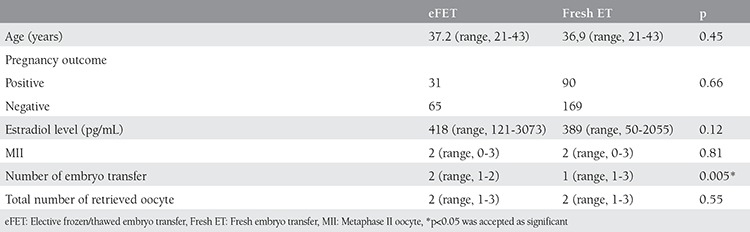
Demographic parameters are displayed. Age, MII oocyte distrubition, total retrieved oocyte number and pregnancy outcomes were similar in both treatment arms but the number of transferred embryos were significantly different

**Table 2:**
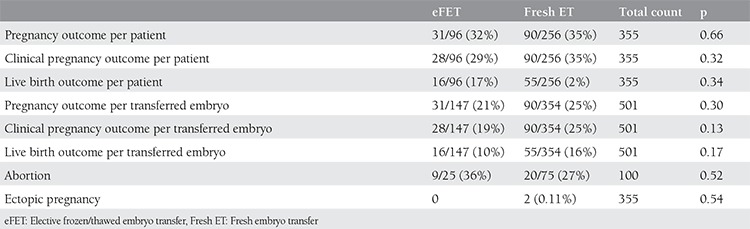
Pregnancy outcomes are displayed. In both treatment arms there was no stastical difference regarding the number of pregnancies, clinical pregnancies, abortion, live births per number of patients, or the number of transferred embryos

**Table 3:**
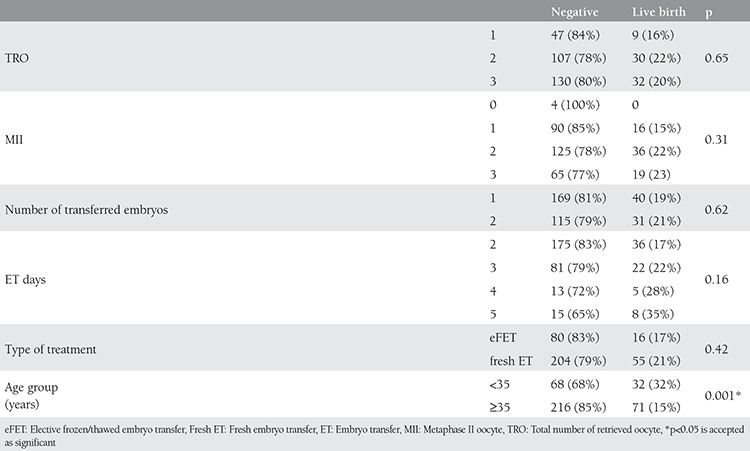
Factors effecting live birth outcomes are displayed. Younger age has increased live birth rate. Total number of retrieved oocyte, number of MII oocytes, type of treatment, number of ET, the day of ET are not effecting live birth rates
